# Falls and visual impairment among elderly residents in ‘homes for the aged’ in India

**DOI:** 10.1038/s41598-020-70066-2

**Published:** 2020-08-07

**Authors:** Srinivas Marmamula, Navya Rekha Barrenkala, Rajesh Challa, Thirupathi Reddy Kumbham, Satya Brahmanandam Modepalli, Ratnakar Yellapragada, Madhuri Bhakki, David S. Friedman, Rohit C. Khanna

**Affiliations:** 1 Allen Foster Community Eye Health Research Centre, Gullapalli Pratibha Rao International Centre for Advancement of Rural Eye care, L V Prasad Eye Institute, Hyderabad, 500034 India; 2 Brien Holden Institute of Optometry and Vision Science, L V Prasad Eye Institute, Hyderabad, India; 3 Department of Biotechnology / Wellcome Trust India Alliance, L V Prasad Eye Institute, Hyderabad, India; 4 School of Optometry and Vision Science, University of New South Wales, Sydney, Australia; 5 Department of Ophthalmology, Massachusetts Eye and Ear, Harvard Medical School, Boston, USA

**Keywords:** Geriatrics, Health services, Public health

## Abstract

We evaluated the prevalence of falls and their association with visual impairment (VI) in elderly residents in ‘homes for the aged’ in Hyderabad, India. Participants aged ≥ 60 years were recruited from 41 homes, and a comprehensive eye examination was conducted. Interviews were conducted to collect personal and demographic information, systemic health status, fear of falling, depression, and history of falls in the last year. VI categories included low vision (presenting visual acuity worse than 6/18 to 3/60) and blindness (presenting visual acuity worse than 3/60). The data of 1,074 participants were analysed. The mean age was 74.4 years (standard deviation:8.7 years); 63.9% were women, 19.4% had no formal education, 28.1% were diabetic and 56.9% were hypertensive. The annual prevalence of falls was 29.1% (95% CI: 26.4–32.0). Multivariable analysis showed those with VI had significantly higher odds of falls (Odds Ratio:1.47; p = 0.043). The prevalence of falls was higher among those with VI due to uncorrected refractive errors. We found a very high prevalence of falls in elderly individuals living in ‘homes for the aged’ in Hyderabad, India. Addressing VI can result in fewer falls and contribute to healthy aging in India.

## Introduction

Falls are common in the elderly, often resulting in catastrophic consequences including pain, loss of independence and poor quality of life^1^. It is estimated that one out of every three elderly individuals report a fall at least once every year of which 20% have serious consequences^2^. The reported prevalence of falls in elderly people in India ranges from about 10% to 53%^3-9^. Falls and their association with visual impairment is reported from large scale epidemiological studies^10,11^. The multi-ethnic Singapore Epidemiology of Eye Diseases (SEED) revealed that participants belonging to Indian ethnicity had a higher risk of falls than those of Malay or Chinese backgrounds^12^.

Most research on falls and the elderly have focused on the community-dwelling elderly population. The lifestyle of the elderly living in residential care and those in the community are not directly comparable. Those living in the community tend to be more mobile and have more physical mobility.^4^ The prevalence of visual impairment is also reported to be higher among the elderly in residential homes compared to those living in communities^13,14^. There are very few studies on falls in the elderly in residential care settings in India. Two studies conducted in Karnataka and Maharashtra state found that poor vision, chronic diseases, poor balance and fear of falling and history of a previous fall as the risk factors for falls in the elderly population^15,16^. Another study from Kerala that included only women found that the prevalence of falls was higher among those in homes for the aged compared to those living in the community^4^. However all the studies mentioned above had a small sample size and limited eye health assessment that is often restricted to vision assessment and/or self-rated vision.

India is witnessing a demographic transition with an increasing proportion of the elderly in the population. Due to societal and lifestyle changes, urbanization and erosion of the traditional joint family system, the numbers of older adults living alone or only with a spouse are rising^17,18^. Another after effect of this breaking down of the traditional support system is resulting in an increase in the number of homes for the aged^17^. Hence a focus on home-based geriatric health care that is comprehensive covering all areas of health including eye care is needed. In this paper, we report on the prevalence of falls, their association with visual impairment and other risk factors among the elderly living in homes for the aged in India.

## Methods

### Study population

The Hyderabad Ocular Morbidity in Elderly Study (HOMES) is a longitudinal study that was conducted during October 2017 and December 2019 among elderly residents (aged ≥ 60 years) living in homes for the aged in Hyderabad in Telangana state in South India. The HOMES study protocols have already been published^19^. This study included 1,182 elderly participants recruited from 41 homes for aged. In our previous publication, we reported a 30.1% prevalence of visual impairment in this population^20^.

### Clinical examination

As a part of the HOMES study, all the elderly participants underwent a comprehensive eye examination including visual acuity assessment, refraction, slit-lamp examination, intraocular pressure measurement, fundus examination, and imaging. The examination procedures have been described in the previous publication^19^. In short, visual acuity (VA) was assessed at a distance of three meters using a log MAR chart under ambient illumination. Both presenting and pinhole visual acuity were assessed. Near vision was assessed using a logMAR chart. The anterior segment was examined using a portable slit lamp. Intraocular pressure was recorded using Perkins Handheld tonometer. A fundus examination and imaging were done for all participants. Visual impairment (VI) was defined using two definitions: 1) Presenting VA worse than visual acuity 6/12 in the better eye, and 2) Presenting VA worse than 6/18 in the better eye. The WHO categories of VI were also used which included low vision (presenting VA 6/18 to 3/60) and blindness (presenting VA worse than 3/60 to no perception of light).

### Non-clinical questionnaires

Trained investigators conducted detailed interviews with the participants using structured questionnaires.^19^ These included an assessment of personal and demographic information (age, education level, marital status, type of home) and systemic history (diabetes and hypertension). The Mini-Mental State Examination (MMSE) was used to assess cognitive status^21^. All the participants with an MMSE score of 20 or more were further interviewed and assessed for depression using the Patient Health Questionnaire (PHQ-9)^22^ and hearing status using the Hearing Handicap Inventory for Elderly (HHIE-S)^23^ questionnaires. Fear of Falling (FOF) was assessed using the Short Falls Efficacy Scale (SFES) questionnaire^24^. This questionnaire had seven questions with the following grades of responses: (1) Not all concerned, (2) Somewhat concerned, (3) Fairly concerned and (4) Very concerned. All the response grades are added to get a cumulative score. Fear of falling is graded as No/low concern (score 7–8), Moderate concern (score 9–13) and High concern (score > 13). In this study, a cumulative score of greater than 8 is considered as having a concern of FOF.

A questionnaire in the local language was used to collect information on the history of falls they had in the last one year. Falls are defined as accidental coming to a halt at the level lower than their normal. The following question was asked, “Have you ever fallen down on the floor in the last one year?” The response was recorded as 0 = No, 1 = Yes and 3 = Cannot remember. All the subjects were also asked to report incidents of falls in the last two weeks. This question was not applicable to the participants who were bedridden. For those who reported a fall within the last two weeks, follow up questions on the consequences of their recent fall and the responses were recorded as (a) No injuries sustained, (b) Had a minor injury that needed no medication nor consultation, and (c) Significant injuries that needed physician consultation.

### Data management

All the data were entered in pre-coded forms and further entered into a database developed in Microsoft Access. Data analysis was conducted using Stata 14.0 (Stata Corp LP, College Station, Tx)^25^. A p-value < 0.05 was considered as statistically significant and two-tailed p values are presented. In the first stage, the prevalence of falls was estimated and presented with 95% confidence intervals (CI). In multiple logistic regression analysis, history of falls was used as an outcome variable and its association with personal and sociodemographic variables (age, gender, education, type of home), systemic conditions (self-report of hypertension and diabetes), depression (PHQ-9) scores^26^. Hearing (HHIE) scores^23^, Short Falls Efficacy Scale (SFES) questionnaire for fear of falling scores^11,24^ and VI as risk factors. Odds ratios (OR) along with 95% CI are presented.

### Data availability statement

The datasets generated during the study are not publicly available as further data analysis is being carried out. The data can be made available from the corresponding author on a reasonable request.

### Ethics approval

The study protocol was approved by the Institutional Review Board of the Hyderabad Eye Research Foundation, L V Prasad Eye Institute, Hyderabad. Each participant provided written informed consent for their participation in the study and was carried out in accordance with the Declaration of Helsinki.

## Results

A total of 1,182 elderly participants were examined out of 1,513 (78.1%) participants enumerated from 41 homes for the aged centres. The mean age of those examined and not examined were not similar (75.0 years versus 74.2 years; p = 0.05). The gender distribution was also similar (64.6% women versus 67.7% women; p = 0.31). Among those examined, 108/1,182 (9.1%) participants were bedridden and hence were not included in the analysis. The data analysis for the prevalence of falls was conducted for the remaining 1,074 participants. (Table 1) The mean age of these participants was 74.4 years (range: 60 – 108 years; standard deviation: 8.7 years). Two-thirds of them were women (63.9%; n = 686), 19.4% (n = 208) with no formal education, 28.1% (n = 302) were diabetic and 56.9% (n = 611) were hypertensive. Among 108 people who were bedridden and not included in the analysis, 11% (n = 12) gave falls as a reason for their current health situation. Other reasons were a general weakness due to old age (n = 60; 55.6%) and other health issues (n = 36; 33.3%).

**Table 1:** Characteristics of the participants and the prevalence of falls in the elderly (n = 1,074) (Univariable analysis).

	Total in the sample	Falls reported		P value ‡
		n	% †	
Age group (years)				0.738
60–69	305	90	29.5	
70–79	418	126	30.1	
80 and above	351	97	27.6	
Gender				0.259
Male	388	105	27.1	
Female	686	208	30.3	
Hypertension				0.594
No	463	131	28.3	
Yes	611	182	29.8	
Diabetes				0.18
No	772	216	28.0	
Yes	302	97	32.1	
Hearing (n = 867) *				0.095
No impairment	682	170	24.9	
Mild-Moderate	126	48	38.1	
Significant	59	24	40.7	
Depression (n = 867) *				< 0.01
Mild/None	672	158	23.5	
Moderate	104	46	44.2	
Severe	91	38	41.8	
Fear of falling (n = 867) *				< 0.01
Low	381	74	19.4	
Moderate	249	73	29.3	
High	237	96	40.1	
Visual Impairment (n = 1,074)				< 0.01
No	773	200	25.9	
Yes	301	113	37.5	
Total	1,074	313	29.1	

* Data not recorded from 207 participants due to poor cognition scores; † Row percentage presented; ‡ p values obtained using the chi-squared test.

**Figure 1:**
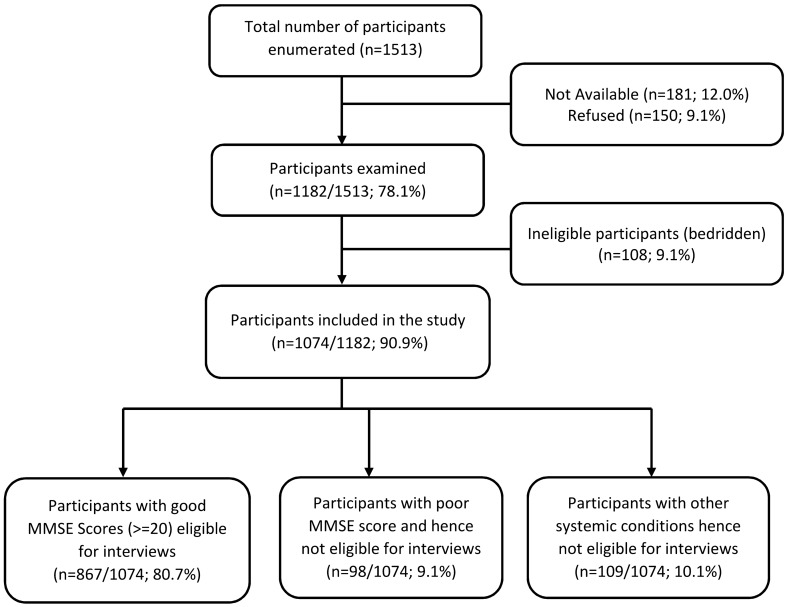
Flowchart showing the study participants.

Of the 1,074 participants, 98 (9.1%) participants had poor MMSE scores and 109 (10.1%) participants had other health issues hence complete interview was not possible. The remaining 867 (80.7%) participants underwent complete interview including administration of PHQ-9 and HHIE questionnaires. (Fig. 1).

### Prevalence and risk factors for falls

Among the 1,074 participants, 313 (29.1%; 95% CI: 26.4–32.0) participants reported a fall in the last year. On univariable analysis, falls had a significant association with depression (p < 0.01), fear of falling (p < 0.01) and VI (definition 1) (P < 0.01). No significant association was found between falls and age, gender, diabetes, and hypertension. (Table 1). On applying multiple logistic regression (model 1), falls were significantly lower among those aged 80 years and older (p = 0.02) and among those with self-reported diabetes (p = 0.03). Those with moderate levels of depression had a higher odds ratio for falls (p < 0.01). Also, those who reported a moderate fear of falling (p = 0.03) and higher concern for falling had a higher odds ratio for falls (p < 0.01). Hypertension and gender were not associated with falls however, mild-moderate hearing impairment was found to associated with falls when VI variable was included in the regression models (Model 2 and Model 3). The odds of falls were significantly higher for both VI definitions. (Table 2).

**Table 2:** Multiple logistic regression assessing the factors associated with falls (n = 867) among the elderly in residential care in India.

	Model 1 (Visual Impairment not included)		Model 2 (Visual Impairment (Definition 1) included)		Model 3 (Visual Impairment (Definition 2) included)	
	Odds Ratio (95% CI)	p value	Odds Ratio (95% CI)	p value	Odds Ratio (95% CI)	p value
Age group (years)						
60–69	Reference		Reference		Reference	
70–79	0.94 (0.65–1.37)	0.75	0.95 (0.65–1.37)	0.77	0.93 (0.64–1.35)	0.71
80 and above	0.61 (0.40–0.94)	0.02	0.60 (0.39–0.93)	0.02	0.59 (0.39–0.91)	0.01
Gender						
Male	Reference		Reference		Reference	
Female	1.2 (0.87–1.66)	0.27	1.21 (0.88–1.68)	0.24	1.20 (0.86–1.66)	0.27
Diabetes						
No	Reference					
Yes	1.44 (1.02–2.03)	0.03	1.49 (1.06–2.10)	0.02	1.49 (1.06–2.10)	0.02
Hypertension						
No	Reference		Reference		Reference	
Yes	0.99 (0.71–1.39)	0.98	1.00 (0.71–1.40)	0.99	0.99 (0.71–1.38)	0.94
Hearing						
None	Reference		Reference		Reference	
Mild-moderate	1.51 (0.98–2.32)	0.06	1.58 (1.04–2.27)	0.03	1.54 (1.04–2.28)	0.03
Severe	1.68 (0.90–2.52)	0.1	2.09 (1.34–3.25)	< 0.01	2.11 (1.36–3.27)	< 0.01
Depression						
None	Reference		Reference		Reference	
Moderate	1.89 (1.19–2.98)	< 0.01	1.92 (1.21–3.04)	< 0.01	1.86 (1.18–2.95)	< 0.01
Severe	1.51 (0.90–2.52)	0.12	1.41 (0.84–2.37)	0.19	1.45 (0.87–2.44)	0.15
Fear of falling						
None	Reference					
Moderate	1.56 (1.06–2.31)	0.03	1.54 (1.00–2.37)	0.04	1.55 (1.01–2.38)	0.04
High	2.15 (1.39–3.34)	< 0.01	1.64 (0.88–3.06)	0.12	1.65 (0.89–3.09)	0.11
Definition 1 – VI*						
No			Reference			
Yes			1.47 (1.01–2.13)	0.043		
Definition 2 – VI ^†^						
No					Reference	
Yes					1.36 (1.00–1.87)	0.05

*Definition 1—Presenting visual acuity worse than 6/18 in the better eye); †Definition 2 – VI—Presenting visual acuity worse than 6/12).

### Falls and categories of visual impairment

The prevalence of falls was 37.5% (95% CI 32.0–43.3) among those who had VI definition 1 (< 6/18 in the better eye) and 32.8% (95% CI 28.9–37.0) among those who had VI definition 2 (< 6/12 in the better eye). Based on the WHO definitions, the prevalence of falls was highest among those with low vision (38.0%; 95% CI 32.2–44.0) followed by those with blindness (33.3%; 95% CI:16.5—54.0) and least among those without VI (25.9%; 95% CI 22.8–29.1). The prevalence of falls also varied with the cause of VI with the highest prevalence of falls among those with VI due to uncorrected refractive errors (46.7% 36.1–57.5). The odds of falls was also higher among those who were visually impaired due to uncorrected refractive errors (OR:2.57 (95% CI:1.48–4.46); p < 0.01) after controlling for other covariates (Table 3).

**Table 3:** Multiple binary logistic regression assessing the factors associated with different definitions of visual impairment (VI), causes of VI and falls (*n* = 867).

	Total in the sample	Falls reported		Odds ratio (95% CI)*	p value
	n	n	Prevalence (95% CI)		
**Definition 1 (Presenting visual acuity worse than 6/18 in the better eye)**					
No visual impairment	773	200	25.9 (22.8–29.1)	Reference	
Visual Impairment present	301	113	37.5 (32.0–43.3)	1.47 (1.01–2.13)	0.043
**Definition 2 (Presenting visual acuity worse than 6/12)**					
No visual impairment	529	134	25.3 (21.7–29.3)	Reference	
Visual Impairment present	545	179	32.8 (28.9–37.0)	1.36 (1.0–1.87)	0.05
**WHO Categories of visual impairment (VI)**					
No visual impairment	773	200	25.9 (22.8–29.1)	Reference	
Low Vision (worse than 6/18–3/60)	274	104	38.0 (32.2–44.0)	1.51 (1.04–2.20)	0.031
Blindness (worse than 3/60)	27	9	33.3 (16.5–54.0)	0.69 (0.13–3.60)	0.66
**Cause-specific VI (< 6/18)**					
No visual impairment	773	200	25.9 (22.8–29.1)	Reference	
Uncorrected Refractive Errors	90	42	46.7 (36.1–57.5)	2.57 (1.48–4.46)	< 0.01
Cataract	134	44	32.8 (25.0–41.5)	1.08 (0.63–1.86)	0.76
Other causes	77	27	35.1 (24.5–46.8)	1.00 (0.48–2.09)	1.00
Total	1,074	313	29.1 (26.4–32.0)		

*Adjusted for age, gender, self-reported diabetes and hypertension, depression, hearing scores, fear of falling scores presented.

### Falls in the last 2 weeks

Based on the response to the questionnaire, 78/1,074 elderly participants reported an episode of falls in the last two weeks (prevalence 7.26%; 95% CI 5.78–8.98). Among these, 16.7% (n = 13) sustained injury and needed to seek consultation with a physician; 32% (n = 25) reported having only a minor injury that needed no consultation; the remaining 51.3% reported no injury. The following reasons were associated with the fall: tripped and fell while walking—78.2% (n = 61); fell while doing household work or self-care—15.4% (n = 12); fell from their cots—6.4% (n = 5).

## Discussion

We found a very high prevalence of falls in the elderly individuals living in homes for the aged in Hyderabad, India. The prevalence of falls was significantly higher among those with low vision. The elderly with uncorrected refractive error had a higher odds of falls even after controlling for all other risk factors. This is an important finding from our study and has far-reaching consequences. Uncorrected refractive error can be corrected with a pair of spectacles and can contribute to fewer falls or prevention of falls in these age groups. The longitudinal nature of the study will provide more insights on the benefit of spectacle correction of reduction in falls as spectacles were provided to all those elderly residents with uncorrected refractive errors. Visual impairment and its association with falls are reported from previous studies^7,27^, such as the Beaver Dam Eye Study and the Blue Mountain Eye Study which reported an association between falls and visual impairment^28,29^.

Also, falls were not associated with cataract even after adjusting for other covariates. This could be due to a lower prevalence of blindness compared to low vision and uncorrected refractive error being the leading cause of low vision. Those with cataract may have a poorer quality of vision and more severe grades of vision loss compared to those with uncorrected refractive errors resulting in limited mobility and a lower risk of falls. Nazlee et al. reported a greater disability and poorer functional measures among those with non-refractive causes of VI compared to that caused due to refractive errors and may have differing consequences^30^. This is also in line with our other finding that those in the 80 years and older age group had a lower odds for falls as this age group tends to have a higher prevalence of cataract related vision loss and often more severe grade of vision loss.

There are several risk factors reported for falls in the elderly^31^. These risk factors include older age, female gender, poor vision, poor gait speed, balance, and cognition^31,32^. While risk factors for falls such as gait disorders or poor balance are consistently reported in studies, other risk factors that are inconsistently reported include visual impairment, cognition issues, hearing loss, and depression^12,15,31,33,34^. Unlike other studies, we did not find any association between gender and falls. The possible reason for this could that our study was done on residents in homes for the aged while other studies have included a community-dwelling elderly population. The proportion of women is higher in homes compared to that in the community. Approximately, 65% of our participants in our study were women. The relationship between falls and fear of falling is reported in a few studies^35-38^. We found a significant association between fear of falling and falls^35-39^. Those who reported a greater fear of falling had higher odds of falling. It is possible that in those with no fear of falling are confident which may act as a defensive mechanism preventing falls. But in the elderly with moderate or high concern of falling, it fails to act a defence mechanism leading to falls.

Depression and its association with visual impairment and falls in the elderly is reported in previous studies^7,40^. We found that moderate depression is strongly associated with falls similar to other studies, however, we did not find any association between severe depression and falls. It can be hypothesized that the elderly with severe levels of depression tend to have limited mobility and consequently a lower risk of falls. Depression, vision loss and falls can act as a triad with a tremendous negative impact on the elderly. Vision loss leading to depression and subsequent falls can be considered as a sequel of events. We hypothesize that this sequel can be broken by intervening to prevent or restore vision which in turn will lead to a decline in depression and subsequent falls. However, there are no studies to prove this hypothesis. We also found that mild-moderate levels of hearing impairment to be associated with falls alongside VI and depression.

As falls in the elderly are multi-factorial a comprehensive approach is needed. From a programme point of view, the risk factors for falls can be categorized into modifiable and non-modifiable risk factors. VI found in the elderly in this study is predominantly avoidable (treatable or correctable) whereas those related to aging cannot be easily reversible. Addressing modifiable risk factors such as VI can help in reducing falls in the elderly. Cataract surgery in one or both eyes is known to prevent falls in elderly^41-43^. However, some studies have shown that cataract surgery is not associated with a reduction in falls in the elderly^44,45^.

Apart from intervention to addressing vision loss, several other interventions can also be implemented to address falls in the elderly. The most common ones include physical exercises for strengthening the lower limbs. Recent studies have shown that doing ‘Yoga’ that includes simple exercises can decrease the rate of falls in the elderly^46^. Newer technology such as machine learning and artificial intelligence impact all domains of our lives with health is no exception. A few researchers have proposed and tested algorithms and mobile technology that can help prevent falls in the elderly^47^.

In this study, we have also collected information on falls experienced by the participants in the last two weeks. A two-week time interval is considered appropriate to minimize recall bias. We found that a large number of elderly reported tripping and falling over while walking. A conducive physical environment can help in preventing falls in the elderly. Having railings in common places can be of immense help to prevent falls. These measures are expected to help those with irreversible vision loss. This becomes even more vital as the number of homes for the aged in India is set to increase in the coming years. We found that only one out of 14 homes that were converted from residences had railings when compared to nine out of 27 in the specially designed homes for the aged. There is a definite need to define the basic minimum infrastructure requirements that should be made mandatory for establishing the homes for the aged or for converting general residences to homes for the aged.

Recall bias is inherent in our study as the elderly may fail to remember the episode of falls and after events especially if the falls were benign and injury-free. This might have led to under-reporting of falls. The lower prevalence of falls in the 80 years and older age group may be attributed partly due to recall issues. It is also possible this elderly group is less mobile due to physical health issues and hence is less prone to falls compared to younger individuals. As in any cross-sectional study design, the issue of survival bias exists in this study also. It is possible that those with severe fall-related injuries are not available in the homes for the aged as they need additional care which cannot be provided in these homes. We also did not collect information on circumstances for falls and step count data which could have provided more insights on physical mobility in the elderly in residential care.

This study focused on the elderly in residential care where only limited research has been carried out. A large sample size with a good response rate and methodological rigor adopted in data collection are the strengths of our study. The results of this study add to the limited information that is available in this domain in India. Given the prediction of an exponential rise in the proportion of elderly people in the coming decades, this research on the elderly becomes highly relevant and has implications on framing health policy for the elderly in India.

The prevalence of falls and their positive association with vision loss, warrants addressing vision loss to minimize falls and risk of falls. The post-intervention phase of this study is expected to provide insights on the magnitude of the impact of the intervention for VI on falls. Falls in elderly is a public health challenge and need to be prioritized for interventions. With simple interventions for vision loss and their positive impact on fall prevention, the cost-effectiveness of vision screening and addressing vision loss in the elderly may be substantial. It is recommended to implement programmes for routine screening for vision loss in the elderly in homes for the aged and it should become as common as school screening programmes and a vital contributing factor towards healthy aging in India.
